# Notes and News

**Published:** 1895-07-13

**Authors:** 


### Notes and News.

(Collected and Communicated.)

The Neurological Society of London held a pathological meeting on July 11th.

The Birmingham Hospital Saturday Fund has now reached £12,768, thus more than fulfilling the expectation of those interested.

The water supply of Paris is about to be added to by utilising the watersprings of the Loing and Lunain Valleys. The expense will be borne by the city of Paris, and will amount to about £1,000,000.

A complimentary dinner was given to Mr. Christopher Heath, the new President of the College of Surgeons, by his old house surgeons. The occasion was chosen for the presentation of a piece of plate in token of their affection and esteem.

The Society for the Employment of Women, in Berners Street, is doing good work. The employments are very varied, comprising hospital nursing and massage, besides clerical work (the largest section), printing, shorthand, chromo-lithography, domestic service, teaching, and numerous others. It is a great advantage to those who have to work for their living that some central agency such as this should be maintained and widely known.

Thanks to the liberality of private individuals the School of Medicine for Women at St. Petersburg is now successfully financed, and all details are arranged. The curriculum is to extend over five years, be the same as that of the medical faculties, and be open to single women over twenty-one years of age. Single women of younger age and married women must be supplied with a written permission from their parents and husbands respectively. All students must know a certain amount of Greek and Latin. A clause provides that a woman holding the school's diploma will not be eligible as director of a general hospital.

Three blind students from the Worcester College for the Blind have passed the recent examination for Responsions at Oxford. This success is unprecedented in the history of the college, from which over forty students have at various times graduated at the Universities.

The new convalescent home at Woodthorpe, in connection with the Nottingham General Hospital, was opened by the Bishop of Derby at the end of last month. The home is the gift of Colonel Seeley, M.P., and cost about £6,000. There are eighteen beds, two of which are for children. A good sized day room is attached to each side of the building, and the whole is beautifully furnished.

The bazaar held in aid of St. Mary's Hospital, and opened by the Princess of Wales on June 27th, proved a great success. The stalls, at which a brisk business was carried on, were prettily decorated, the young ladies assisting stall-holders being most becomingly dressed. A number of excellent entertainments added to the attractions of the proceedings. The proceeds, we are informed, will reach quite £5,000.

The Commission on Leprosy in India has concluded its work. The Commissioners have arrived at the conclusion that the alarm at the spread of leprosy in India is groundless, for they find that the progress of the disease has decreased. Many interesting facts and statistics have been brought to light during the holding of the Commission, towards the expenses of which the National Leprosy Fund have devoted large sums.

The Marquis of Dufferin makes the following appeal on behalf of the Royal Free Hospital: "As President of the Royal Free Hospital I have the honour to announce that their Royal Highnesses the Prince and Princess of Wales have graciously consented to open the new front building of the hospital on Monday, July 22nd. The cost of the new buildings and of various other improvements which have been carried out during the past two years amounts to £30,000, towards which sum £24,000 has already been raised. I now ask your favourable consideration to the enclosed appeal of the committee, feeling assured that I may rely upon your generosity to assist them in obtaining the sum of £6,000, which is still required to enable them to carry on the benevolent work of the hospital free from debt."

It has been decided to establish, in connection with the Charing Cross Hospital Medical School, a permanent memorial to one of its most distinguished students, the late Professor Huxley. To this end the following committee has been formed: Sir Joseph Fayrer, K.C.S.I., F.R.S., Sir Guyer Hunter, K.C.M.G. (both old friends and fellow-students of Professor Huxley at the Charing Cross School), Dr. Watt Black (hon. treasurer), Mr. J. H. Morgan, Mr. Stanley Boyd, Dr. Montague Murray, and Mr. H. F. Waterhouse (hon. secretary). It is proposed that the memorial shall take the form of an annual lecture and a science scholarship and medal; but the final decision will depend upon the wishes of a general meeting of the subscribers. Subscriptions will be received and acknowledged by Dr. Watt Black, at the Charing Cross Hospital Medical School.

